# On the functional role of striatal and anterior cingulate GABA+ in stimulus‐response binding

**DOI:** 10.1002/hbm.25335

**Published:** 2021-01-09

**Authors:** Adam Takacs, Ann‐Kathrin Stock, Paul Kuntke, Annett Werner, Christian Beste

**Affiliations:** ^1^ Cognitive Neurophysiology, Department of Child and Adolescent Psychiatry, Faculty of Medicine TU Dresden Dresden Germany; ^2^ Biopsychology, Department of Psychology, School of Science TU Dresden Dresden Germany; ^3^ Institute of Diagnostic and Interventional Neuroradiology TU Dresden Dresden Germany

**Keywords:** anterior cingulate cortex (ACC), gamma‐aminobutyric acid (GABA), magnetic resonance spectroscopy (MRS), response selection, striatum, theory of event coding (TEC)

## Abstract

Successful response selection relies on constantly updating stimulus–response associations. The Theory of Event Coding (TEC) proposes that perception and action are conjointly coded in event files, for which fronto‐striatal networks seem to play an important role. However, the exact neurobiochemical mechanism behind event file coding has remained unknown. We investigated the functional relevance of the striatal and anterior cingulate (ACC) GABAergic system using magnetic resonance spectroscopy (MRS). Specifically, the striatal and ACC concentrations of GABA+ referenced against N‐acetylaspartate (NAA) were assessed in 35 young healthy males, who subsequently performed a standard event file task. As predicted by the TEC, the participants' responses were modulated by pre‐established stimulus response bindings in event files. GABA+/NAA concentrations in the striatum and ACC were not correlated with the overall event binding effect. However, higher GABA+/NAA concentrations in the ACC were correlated with stronger event file binding processes in the early phase of the task. This association disappeared by the end of the task. Taken together, our findings show that striatal GABA+ levels does not seem to modulate event file binding, while ACC GABA+ seem to improve event file binding, but only as long as the participants have not yet gathered sufficient task experience. To the best of our knowledge, this is the first study providing direct evidence for the role of striatal and ACC GABA+ in stimulus–response bindings and thus insights into the brain structure‐specific neurobiological aspects of the TEC.

## Graphical abstract

The Theory of Event Coding (TEC) proposes that perception and action are conjointly coded in event files, for which fronto‐striatal networks seem to play an important role, but the exact neurobiochemical mechanisms behind even file coding have remained unknown. We investigated the functional relevance of the striatal and anterior cingulate (ACC) GABAergic system using magnetic resonance spectroscopy (MRS) in thirty‐five adults, who subsequently performed a standard event file task. GABA+/NAA concentrations in the striatum and ACC were not correlated with the event binding effect across the entire task, but higher GABA+/NAA concentrations in the ACC were correlated with stronger event file binding processes in the early phase of the task.

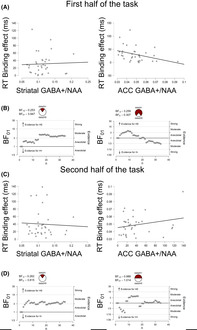

## 1 INTRODUCTION

Adapting our reactions and responses to an ever‐changing environment is a major achievement. It is particularly challenging when new information needs to be integrated, as this requires the reconfiguration of pre‐established stimulus–response associations. The Theory of Event Coding (TEC) (Hommel, 2004; Hommel, Müsseler, Aschersleben, & Prinz, 2001) assumes that perception and action share common processing codes, where representations of actions include their perceptual consequences, and vice versa. The TEC postulates that whenever we select an action, associated stimulus features also become activated. This suggests that response selection is not an isolated process, and should therefore be studied in the context of perception and action integration (Hommel, 2009, 2011; Moeller, Pfister, Kunde, & Frings, 2019).

The cognitive representations of objects (so‐called “object files”), contain information about a given object's features, such as colour, orientation, size, etc. (Treisman & Kahneman, 1984), while planning a response evokes a so‐called “action file” containing the relevant features of the given action (Hommel, 2004; Hommel et al., 2001). During the integration of perception and action, the codes of object files and action files undergo a complex, multi‐layered code sharing/binding process that gives rise to so‐called “event files” (Hommel, 2004; Moeller et al., 2019). These binding processes can be studied based on the finding that temporal co‐occurrence induces binding between a stimulus and a response. The resulting event file will be (re‐)activated once a single feature of a stimulus, or a response, is (re‐)encountered (Frings et al., 2020; Hommel, 2011). This has important consequences for the efficacy of response selection and response execution in event‐file coding paradigms, where participants are asked respond to a stream of stimuli that vary in how many object features they share with the preceding stimulus: Whenever identical or similar stimuli require different responses, the previously established event file bindings cause problems as they are only partially fulfilled (Colzato, Warrens, & Hommel, 2006; Hommel, 2004). In other words, similar or identical stimuli (re‐)activate the same response that was previously carried out, which leads to conflict and the need for reconfiguration whenever a different response would be correct. Importantly, the required reconfiguration hampers behavioural performance by slowing down responses and increasing error rates, which is termed “partial repetition costs” (Colzato, Warrens, et al., 2006; Hommel, 2004). In contrast to this, but following the same functional principle, pre‐established event file bindings facilitate responding whenever identical or similar stimuli require the same response(s). Thus, event files can both improve or hamper general response selection mechanisms through the binding and retrieval of stimulus–response associations (Frings et al., 2020).

A growing number of studies have investigated the neural correlates of event file coding, using various methods from functional magnetic resonance imaging (Kühn, Keizer, Colzato, Rombouts, & Hommel, 2011), EEG time‐frequency (Keizer, Verment, & Hommel, 2010; Keizer, Verschoor, Verment, & Hommel, 2010) and time‐domain analyses (Opitz, Beste, & Stock, 2020; Pastötter & Frings, 2018; Petruo, Stock, Münchau, & Beste, 2016; Takacs, Zink, et al., 2020), to decoding of neural activity (Kikumoto & Mayr, 2019; Takacs, Mückschel, Roessner, & Beste, 2020). Importantly, a number of studies have also examined the neurobiochemical underpinnings of event file coding processes, mostly using pharmacological (Colzato et al., 2012) or molecular genetic methods (Colzato et al., 2016; Persson, Rieckmann, Kalpouzos, Fischer, & Bäckman, 2015). Although these studies provide valuable insights, they cannot account for brain structure‐specific effects of the investigated neurobiochemical factors. Yet, this is crucial because several brain regions, including fronto‐striatal circuits, are very important for response selection (Chudasama & Robbins, 2006). These include the medial frontal cortex and the anterior cingulate cortex (ACC), which play a key role for response selection (Dalley, Cardinal, & Robbins, 2004; Rushworth, Buckley, Behrens, Walton, & Bannerman, 2007; Shenhav, Straccia, Musslick, Cohen, & Botvinick, 2018). Within the ACC, the gamma‐aminobutyric acid (GABA) system has been demonstrated to modulate response selection and control processes (Silveri et al., 2013). Given that event coding processes also appear to be associated with anterior cingulate structures (Petruo et al., 2016), it is possible that variations in the ACC GABA system may predict inter‐individual differences in event file processing and binding dynamics. As this has never been tested, we set out to investigate that question. However, the function of the ACC cannot be accounted for without considering closely connected basal ganglia structures that also contribute to response selection and also heavily depend on GABA, like the striatum (Chudasama & Robbins, 2006; Middleton & Strick, 2000; Parvizi, 2009).

GABAergic medium spiny neurons (MSNs) make up the majority of striatal cells (Bolam, Hanley, Booth, & Bevan, 2000). Neighbouring striatal MSNs form a dense inhibitory feedback network (Tunstall, Oorschot, Kean, & Wickens, 2002), thereby creating a complex “winner‐take‐all” network (Plenz, 2003). Computational accounts suggest that this GABAergic winner takes all network is central for response selection (Beste, Humphries, & Saft, 2014; Gurney, Prescott, & Redgrave, 2001; Gurney, Prescott, Wickens, & Redgrave, 2004; Humphries, Stewart, & Gurney, 2006; Redgrave, Prescott, & Gurney, 1999; Tomkins, Vasilaki, Beste, Gurney, & Humphries, 2014): It was proposed that competing (stimulus or response) feature codes inhibit each other during event file coding, which then leads to “winner‐take‐all” processing (Kühn et al., 2011). In line with this, some studies suggested that striatal structures play a key role in event file coding (Colzato et al., 2016; Persson et al., 2015). Further adding to this, other studies suggest that striatal GABA levels can predict performance in response selection and control processes (Haag et al., 2015; Quetscher et al., 2015; Yildiz et al., 2014). Therefore, the GABA systems in both the ACC and striatum may predict behavioural dynamics of event file coding. We hypothesized that if higher GABA levels facilitated the selection of predominant responses through winner‐take‐all processing, this positive link should also be evident in behavioural measures of event file coding. Specifically, higher GABA levels should be related to stronger event file binding, and therefore stronger stimulus–response associations, which are reflected by higher partial repetition costs.

Additionally, the striatal GABAergic system may also be important for how event files are established and processed. The underlying binding between perception and response is established rather automatically (Hommel, 2005) and constitutes a memory trace of the given event (Hommel, 2011). It has been demonstrated that gamma synchronization is related to recollection of episodic memories, retrieval of event files, and intelligence (Keizer, Verment, et al., 2010; Keizer, Verschoor, et al., 2010), suggesting a common underlying mechanism. Furthermore, control over memory trace retrieval is largely dependent on the suppressing and gating activity of the ACC (Anderson, Bunce, & Barbas, 2016). Hence, event file coding might depend on the strength of associations between stimuli (object files) and responses (action files), as the partial repetition costs are particularly high when (strong) pre‐established bindings persist.

Since the GABAergic system seems to play an important role for response selection, it is conceivable that the planned correlation analysis reveals a different pattern as the task progresses: The striatum and especially the striatal GABAergic system have repeatedly been suggested to play a role in acquiring S‐R associations (Freedberg, Toader, Wassermann, & Voss, 2020; Graybiel & Grafton, 2015; Kreitzer & Malenka, 2008; Miyachi, Hikosaka, Miyashita, Kárádi, & Rand, 1997; Perrin & Venance, 2019; Schumacher, de Vasconcelos, Lecourtier, Moser, & Cassel, 2011). Therefore, the functional role of striatal GABA might be different for event file coding in early and late phases of the task. Similarly, the role of the ACC GABAergic system does not necessarily have to be uniform throughout the task, as striatal and prefrontal systems compete for dominance in information processing (Daw, Niv, & Dayan, 2005; Filoteo, Lauritzen, & Maddox, 2010). Specifically, GABA levels might predict task performance in novel situations (i.e., at the beginning of task performance), when response selection tendencies still need to be established. Later, this relationship could disappear once memory traces of the events are sufficiently stabilized.

Aside from the functional role of striatal and ACC GABA in adaptive response selection, it should however not be forgotten that the dopaminergic system has often been considered to be equally, if not more important than the GABAergic system for keeping task representations “online” (Durstewitz & Seamans, 2008; Seamans & Yang, 2004). This notion is also supported by studies showing that modulations in the dopaminergic system affect event file binding processes (Colzato et al., 2012, 2016; Colzato, Zmigrod, & Hommel, 2013). As event file binding may thus be modulated by GABA and / or dopamine, it is also potentially possible that event file binding processes are not significantly modulated by the GABAergic system. If this was to hold true, there should be no correlations between striatal and/or ACC GABA levels and event file binding processes.

In summary, we investigated whether or not striatal/ACC GABA levels modulate event file binding due to their effects on response selection via winner‐takes‐all mechanisms.

## 2 METHODS

### 2.1 Participants

Previous studies revealed substantial effects sizes (R^2^ = .40) in correlations between striatal GABA+ levels, as measured using magnetic resonance spectroscopy (MRS), and behavioural indices of cognitive control processes (Haag et al., 2015; Quetscher et al., 2015; Yildiz et al., 2014). A priori power calculations (using the G*Power software package; Faul, Erdfelder, Buchner, & Lang, 2009) revealed a required a sample size of *n =* 35 to achieve a power of 95% with an alpha error probability of 5% (given R^2^ = .40). Therefore, *n* = 35 healthy male participants (23.4 ± 3.8 years of age) were recruited for the study. Females were not included as changes in steroid hormones across the menstrual cycle have been suggested to modulate GABA levels (Epperson et al., 2006; Harada, Kubo, Nose, Nishitani, & Matsuda, 2011). All participants reported no history of neurological or psychiatric disorders and had normal or corrected‐to‐normal vision. All participants were undergraduate or graduate students and received a financial reimbursement of 20 € for their participation. All participants gave written informed consent prior the measurements. The study was conducted in accordance with the declaration of Helsinki and approved by the ethics committee of the TU Dresden. Lastly, please note that most of the participants included in this sample were also included in the sample of a previous publication by our group (Bensmann, Zink, Werner, Beste, & Stock, 2020; Stock, Bensmann, Zink, Münchau, & Beste, 2019).

### 2.2 Task

**FIGURE 1:**
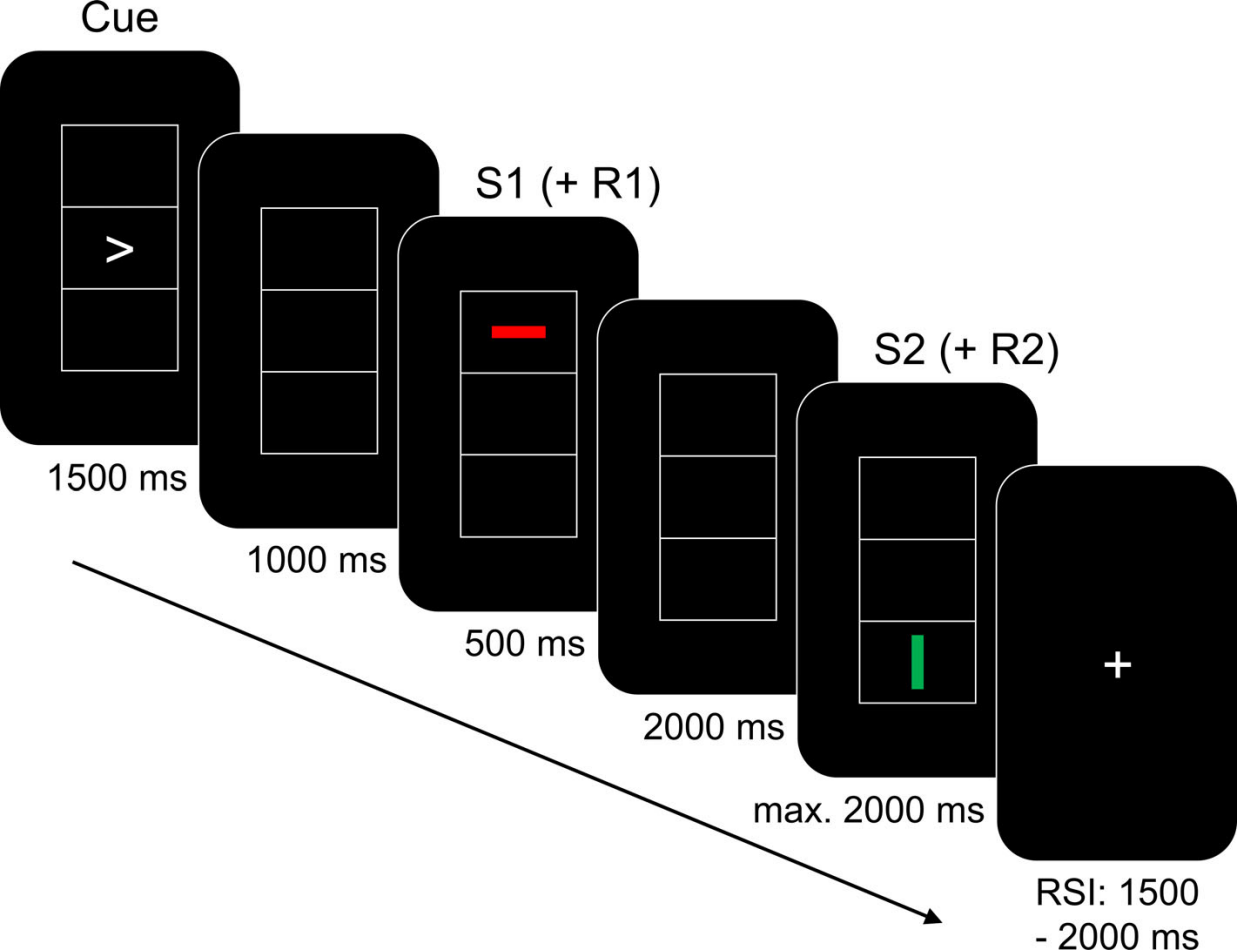
Schematic illustration of the event file coding task. The figure represents the order of the stimuli during a trial. The first response (R1), which was required to indicate the pointing direction of the cue stimulus, had to be given during the presentation of the S1 stimulus. The second response (R2), which was required to indicate the orientation of the S2 stimulus, had to be given during the presentation of that same S2 stimulus and ended its presentation

Stimulus‐response binding was examined with a standard event file coding paradigm developed by Colzato, Warrens, et al., 2006 in the version recently used by Stock et al. (2019) and Kleimaker et al. (2020). The task is depicted in Figure 1.

Participants were seated at a distance of 60 cm from a 17‐in. CRT screen. During the experiment, they saw three vertically aligned boxes, each of them sized 2.8 × 2.2 cm. This triad of boxes was presented in the centre of the screen. In the middle box, participants first saw a response cue (“>” or “<”) pointing either left or right. This was followed by the consecutive presentation of two single bar stimuli of 1.2 × 0.3 cm. Each of these bars could be oriented either vertically and horizontally (task‐relevant feature of orientation), be either red or green (task‐irrelevant feature of colour), and be placed in either the top or the bottom box of the visual array (task‐irrelevant feature of location). Based on the time of their occurrence, these lines served as stimulus 1 (S1) and stimulus 2 (S2). Importantly, each combination of all three stimulus features across S1 and S2 occurred equally often so that S1 stimulus features could not serve as a predictor of S2 stimulus features. As a consequence of this variation, S1 and S2 could share between zero and three stimulus features: Trials could have none of the three features shared between S1 and S2 (no feature overlap condition), one or two shared features (partial overlap conditions), or all three features shared between S1 and S2 (full feature overlap condition). Participants had to execute two responses per trial (R1 and R2) by pressing the left or right control key of a computer keyboard with the corresponding index fingers. As a result, the two consecutive responses could require either response repetition (R1 and R2 identical) or response alternation (R1 and R2 on opposite sides and thus executed with opposite fingers). The task was designed to study binding effects, that is, the functional interaction between repetitions of stimulus features (through varying feature overlap between S1 and S2) and responses (through varying motor response overlap between R1 and R2). The timing of the experiment was the following in every trial: First, the cue appeared on the screen for 1,500 ms. Participants were instructed not to react immediately to the cue, but withhold their response until the presentation of S1. After the response cue, a blank screen was displayed for 1,000 ms. It was followed by the S1 with a duration of 500 ms. During the presentation of the S1, participants had to execute R1 (right keypress in case the cue arrowhead had been pointing right, and left keypress in case of a left‐pointing arrowhead). Importantly, each combination of cue and S1 properties appeared equally often so that the R1 was entirely unrelated to any of the S1 stimulus features (orientation, colour, location). Despite the irrelevance of S1 features, the temporal overlap of S1 and R1 still creates an association (binding) between S1 and R1. Whenever participants failed to press the correct R1 response button during S1 presentation, the trial was aborted and started anew for up to 3 times in a row in order to ensure proper temporal coupling and thus the proper binding of S1 and R1 in an event file. When participants had managed to carry out a correct R1 response during S1 presentation (or when the combination of cue and S1 had been repeated for 3 times), the S1 was followed by a blank screen for 2000 ms. Next, the S2 was presented until a R2 response was given, but no longer than 2000 ms. Participants were required to respond to the orientation of the S2 by pressing the left key when the S2 was oriented horizontally and the right key when the S2 was oriented vertically. The whole session comprised 256 trials in four equally sized blocks and lasted about 35 to 40 min without practice. During inter‐trial intervals, which were jittered between 1,500 and 2,000 ms, a fixation cross was presented in the centre of the screen.

### 2.3 MRS data acquisition and processing

**FIGURE 2:**
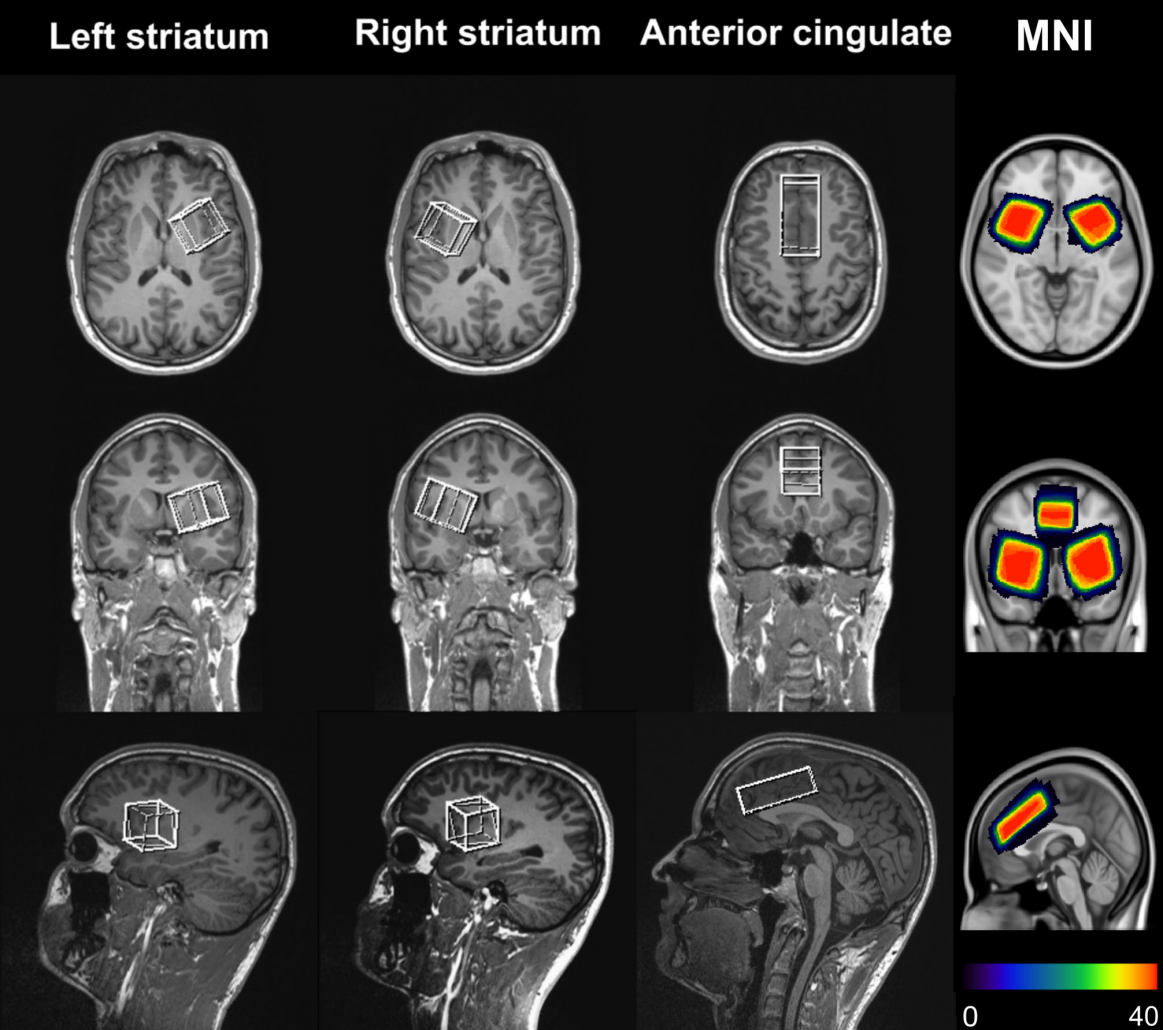
Illustration of the volume of interest (VOI) placements in the striatum, and the ACC (please note that sides are mirrored in this scanner output). For the striatum, the 30 × 30 × 30 mm VOIs were positioned in three consecutive steps, which were comparable for both sides. First, the VOI was aligned along the three axes and positioned at the anterior dorsal striatum to cover the caudate and putamen. Then, the VOI was rotated medially/“inwards” towards the midline on the axial plane (top row) to cover as much as possible of the caput of the caudate nucleus. Next, the lateral side of the VOI was rotated dorsally in the coronal view (middle row) to cover as much as possible of the dorsal caudate nucleus. Then, the VOI was rotated in the anterior direction in the sagittal view (bottom row) to cover as much as possible of the anterior caudate and the putamen. Finally, the positioning was inspected again in all three views. If it was necessary, the positioning was adjusted again to make sure that the VOI did not cover the lateral ventricles or other brain structures containing larger amounts of free cerebrospinal fluid. For the ACC, the 20 × 60 × 30 mm VOI was positioned in three consecutive steps. In the beginning, the VOI was aligned along the three axes and positioned at the ACC to cover as much as possible of this structure without including any parts of the ventricles. In the sagittal view (bottom row), the anterior side of the VOI was rotated ventrally while the VOI was shifted along the anterior–posterior and ventral‐dorsal axes until the ventral border of the ACC VOI aligned with the anterior dorsal border of the corpus callosum and the anterior dorsal edge of the ACC VOI reached the anterior border of the ACC. Next, the VOI was moved laterally in the axial view (top row) until the sagittal centre aligned with the brain's midline. If necessary, the VOI was further adjusted not to cover the lateral ventricles in the coronal view (top row). The rightmost column depicts the size and position of each participant's VOIs in an MNI template overlay map. The resulting heat maps represent the average positioning, with warmer colours denoting more overlap between the individuals' positioning (i.e., zero participants overlapping is denoted by dark purple while the overlap of all 35 participants is denoted by reddish orange; compare colour bar in the bottom right corner). An animated 3D illustration of the overlap is also provided in Video S1

MRS data acquisition and processing followed the protocol previously described by Bensmann et al. (2020): We measured GABA+ concentrations in the striatum and the ACC using 1H‐MR‐spectroscopy. Structural MRI and MRS data were acquired using a 3T Prisma scanner (Siemens Healthineers, Erlangen, Germany) with a 32‐channel (receive only) headcoil. After the localizer, a high‐resolution 3D T1‐weighted sagittal Magnetization Prepared Rapid Gradient Echo (MPRAGE) sequence (1 mm isovoxel) was conducted and reconstructed for exact voxel placements. Voxels of interest (VOIs) were placed in the ACC (20 × 30 × 60 mm, over the midline) as well as in the left and right striatum (30 × 30 × 30 mm), to rule out any laterality effects. The positioning of the VOIs is depicted in Figure 2.

The selection and placement of VOIs were in accordance with previous studies investigating striatal GABA+ levels in response selection processes (Marjańska et al., 2013; Tremblay et al., 2014). Please note that the ACC VOI covered large parts of the dorsal ACC and included only small fractions outside of the ACC. However, the striatal VOIs also included considerable amounts of adjacent structures. This was inevitable, as a sufficiently large voxel of 3 × 3 × 3 cm is required/recommended for a reliable quantification of GABA+ (Mikkelsen et al., 2017) and smaller VOIs covering less or none of the adjacent structures would have resulted in insufficient signal quality. Against this background, our striatal VOI placement aimed to include as much as possible of the anterior and dorsal striatum, because the caudate is likely the striatal structure that is most important for response selection processes. The putamen, which was also included in the striatal VOIs, receives motor and sensory inputs, and as such, is likely also involved in event file binding. As a consequence, the ventral and posterior parts of the striatum were usually not completely included in the VOIs, but given that the ventral striatum is mainly characterized by limbic inputs, it was not of primary interest in the current study (Gerfen & Bolam, 2010; Iversen, 2010; Yildiz et al., 2014). To generate overlay maps illustrating the adequate positioning of VOIs, the size and position of the VOIs were extracted from the rda‐files (raw data) and exported as 3D mask files using the Gannet software (http://www.gabamrs.com/) (Edden, Puts, Harris, Barker, & Evans, 2014). Using the “antsRegistrationSyNQuick.sh” tool from Advanced Neuroimaging Tools (Avants et al., 2011), the 3D T1‐weighted images of all participants were registered to the MNI152 (1 mm) space. The resulting affine matrix and nonlinear warpfield were then used to warp the voxels into the MNI‐space. After repeating the process for each participant, the results of all participants were overlaid to show the quality of voxel positioning (see Figure 2). To further address this issue, we additionally provide an animated 3D depiction (created with the “PARAVIEW” software tool; https://www.paraview.org/), to demonstrate the voxel positions and overlay from different angles (see Data S1).

In addition to the inbuilt shim routine, manual shimming was performed to further improve / optimize spectral resolution by obtaining a full width at half maximum (FWHM) value below 20 Hz for the unsuppressed water signal. MRS data were acquired using the CMRR (Center for Magnetic Resonance Research) MEGA‐PRESS (Mescher‐Garwood point‐resolved spectroscopy) sequence (echo time TE/ repetition time TR = 68/2000 ms, edit ON acquisitions = 128, edit OFF acquisitions = 128) developed by Edward J. Auerbach and Małgorzata Marjańska and provided by the University of Minnesota (Marjańska et al., 2013; Tremblay et al., 2014), based on a C2P licence agreement with Siemens Healthineers AG Germany. Additionally, a SVS_se30 sequence (echo time TE / repetition time TR = 30/ 2000) was acquired with water suppression (NS: 128) and without water suppression (NS:16; for eddy current corrections) to better account for metabolites with short T2‐values, like glutamate (Glu) and glutamine (Gln), which were summarized as Glx (please refer to the Data S1 for results on this).

**FIGURE 3:**
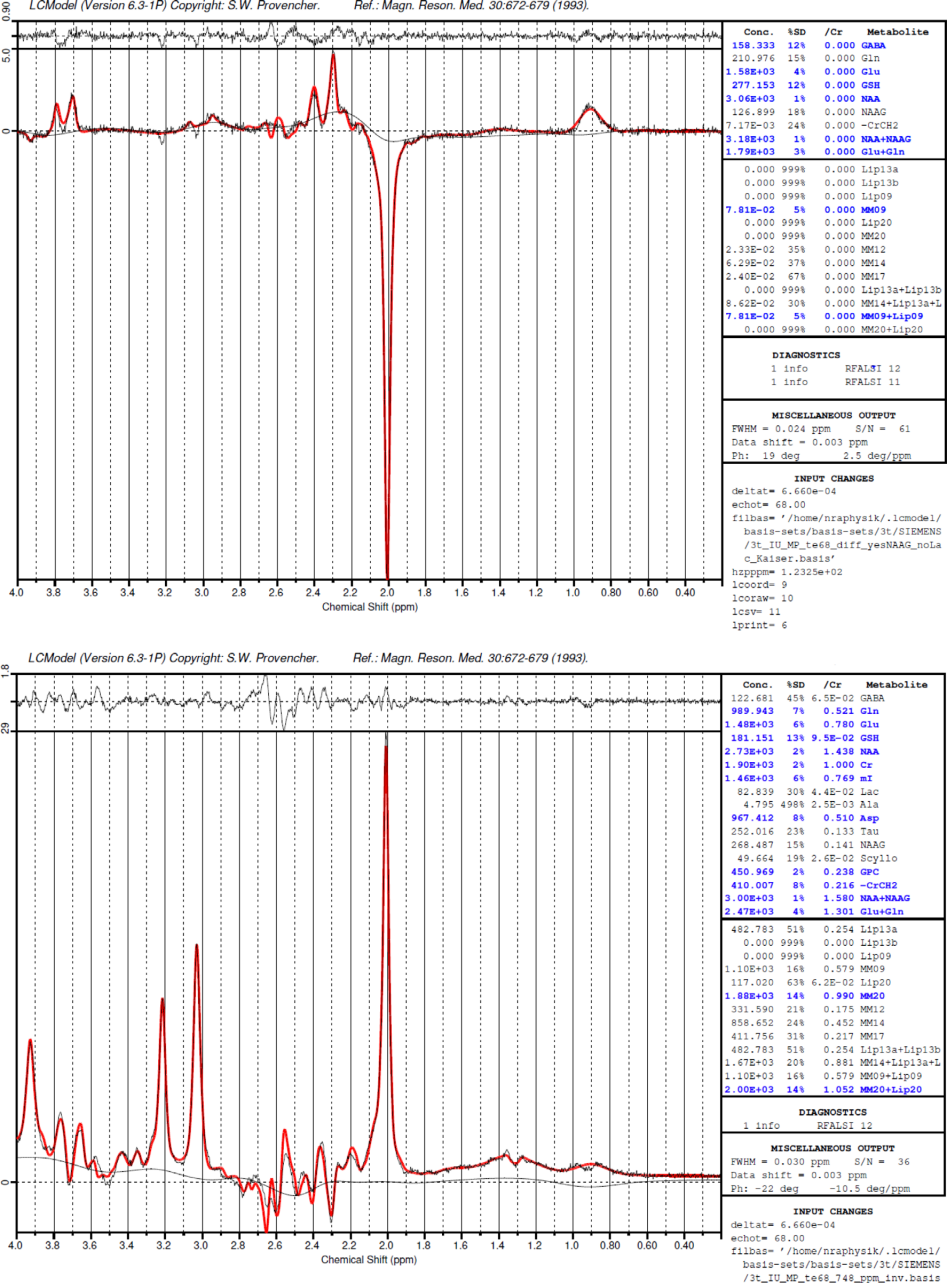
Representative example of the MEGA‐PRESS edited fitted spectrum, metabolite estimates (in arbitrary units, on the right side), and the residuals (top rows of the graphs). The grey line represents the measured spectrum and the baseline. The red line shows the LCModel fit. The upper panel shows the GABA+ difference spectrum (*“edit on” ‐ “edit off”*), the lower panel shows the “edit off spectrum”

**FIGURE 4:**
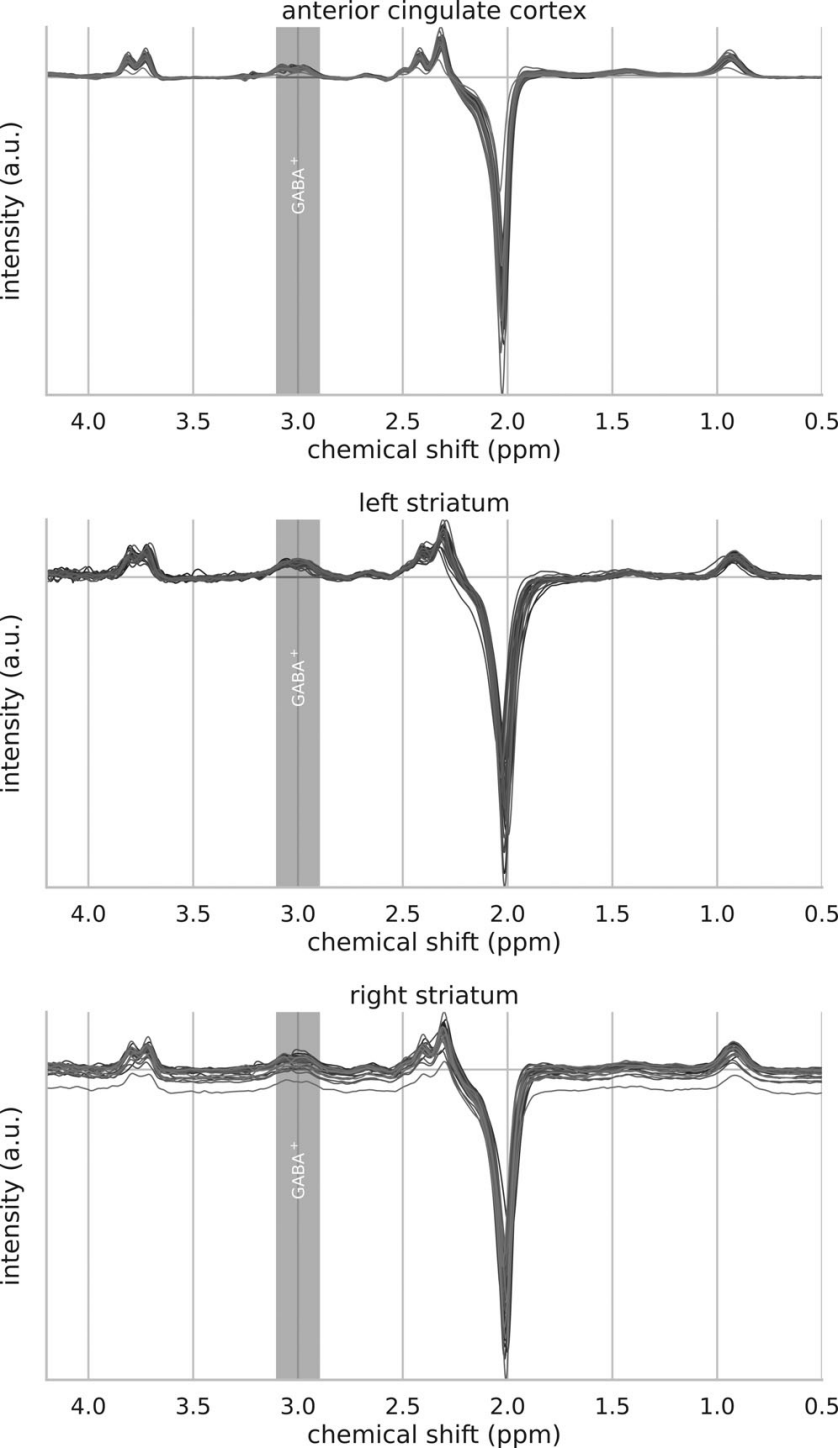
Overlay of each participant's spectra for all three VOIs. The grey bar highlights the GABA peak(s)

LCModel software (v6.3‐1H) (Provencher, 1993), which fits the in vivo MR spectra as a linear combination of single metabolite basis spectra, was used to quantify the obtained spectra. Basis sets for MEGA‐PRESS, generated from density matrix simulations of the sequence, were delivered by Ulrike Dydak's Lab at Purdue University (http://purcell.healthsciences.purdue.edu/mrslab/basis_sets.html). For the quantitation of GABA+, we the used the “3T Siemens Difference Basis Set with Kaiser Coupling Constants”, based on updated values for chemical shifts and J‐GABA coupling constants (Kaiser et al., 2008; Kreis & Bolliger, 2012; Near, Evans, Puts, Barker, & Edden, 2013) (these slightly differ from to the originally generated basis sets by Dydak et al., 2011, which used the values by Govindaraju, Young, & Maudsley, 2000). Based on the “edit off” spectra from the same MEGA‐PRESS measurement and using the corresponding “3T Siemens Edit‐off Basis set”, total creatine (*t*Cr) and N‐acetylaspartate (NAA) reference values for GABA+ were estimated. With regard to the subsequent quantification of MRS spectra, only spectra of final adequate shim quality (FWHM of 3–7 Hz of the NAA peak) were used to ensure sufficient data quality. Representative LCModel fits of MEGA‐PRESS are depicted in Figure 3. An overlay of each participant's spectra for all three VOIs is provided in Figure 4.

A 20% Cramér‐Rao lower bound (CRLB or %SD) criterion was used on the absolute GABA+ error estimate (Kreis & Bolliger, 2012). This led to the exclusion of *n* = 1 participant from the correlation analyses. In the remaining sample, the CRLB was adequate for analysing the data from the striatum (right side: 11.75 ± 2.04, left side: 10.86 ± 1.97) and from the ACC (12.34 ± 2.49). Of note, absolute GABA+ values are not optimal for the planned investigation of correlations between behavioural parameters and GABA levels in the striatum and ACC. We decided to address this issue with the help of an internal reference signal (Mikkelsen et al., 2019). GABA+ is often referenced to *t*Cr or NAA (Mikkelsen et al., 2017, 2019), but the use of an internal reference is only valid if the reference itself does not have a systematic relationship with the neurotransmitter of interest and/ or the other studied (in our case behavioural) parameters (Mikkelsen et al., 2019). In our dataset, *t*Cr levels significantly correlated with the main behavioural binding measure of the study (see Section 2.4) in the ACC (*r* = −.363, *p* = .034, *BF*_01_ = 0.55), but not in the striatum (*r* = −.185, *p* = .414, *BF*_01_ = 2.77). In contrast to this, NAA levels were not correlated with the binding effect (striatum: *r* = −.220, *p* = .212, *BF*_01_ = 2.22; ACC: *r* = −.286, *p* = .100, *BF*_01_ = 1.29). Therefore, we decided to use the ratio of GABA+ and NAA for all subsequent analyses of GABA concentrations in the striatum and ACC. Furthermore, the concentrations of the different metabolites were averaged across the left and right striatal VOIs and then used to form a composite GABA+/NAA ratio for all statistical analyses. This was not necessary for the ACC, since the voxel had already been placed at the midline and therefore covered both the left and right ACC. To complement the analyses of GABA+/NAA, we also report a composite ratio of Glx (sum of glutamate and glutamine) and NAA, and the ratio of GABA+ and Glx in Data S1.

As already mentioned, different proportions of WM, GM and CSF in MRS voxels of the striatum and ACC may potentially also influence the metabolite levels. While we had chosen to address this issue by means of an internal reference, we additionally quantified the fractions of these three types of tissue. The registration and segmentation functions in Gannet toolkit (http://www.gabamrs.com/) (Edden et al., 2014) were applied to quantify the fractions of GM, WM and CSF volumes within the three VOIs. After the fractions of these three components had been calculated, they were averaged for both sides of the striatum (analogously to the averaging procedure for metabolites described above). As the fractions of GM, WM, and CSF did not correlate with the levels of GABA+, NAA, and GABA+/NAA in the ACC (all *p* ≥ .349) or in the striatum (all *p* ≥ .225), we refrained from controlling for these factors in the following analyses.

Lastly, all MR‐spectroscopy measurements were conducted at about the same time (in the afternoon), as GABA+ levels are known to be subject to circadian changes (higher concentrations at night) (Marquez de Prado et al., 2000). The average time difference between the behavioural task and MRS acquisition was less than 1 day (0.5 days ± 2.3).

### 2.4 Statistics

Statistical analyses were performed using JASP 0.11.1 (JASP Team, 2019). Error rates (percentage of incorrect responses for S2) and median S2 response times (RTs) of correct trials were obtained for each participant and each condition.

To examine event file coding, error rate and RT data were analysed in separate two‐way repeated measures ANOVAs with feature overlap (no, one, two, or full feature overlap) and response type (repetition vs. alternation) as within‐subject factors. This approach follows statistical procedures of previous studies examining binding effects in the event coding framework (Petruo et al., 2016; Stock et al., 2019). We report partial eta square (η_p_^2^) effect sizes for ANOVA main effects and interactions. All post‐hoc tests were Bonferroni‐corrected.

To examine the relationship between the assessed behavioural and neurobiochemical data, Pearson correlations were conducted. Specifically, RT differences between full feature overlap and zero overlap were calculated separately for response repetition and response alternation. In the next step, the absolute values of these differences were averaged. This was necessary, as partial repetition costs and repetition benefits can both reflect the strength of binding, even though they affect response time and accuracy in opposite directions: Due to the partial repetition cost (Colzato, Warrens, et al., 2006; Hommel, 2004), responses are slower and the error rate is higher whenever identical or similar stimuli necessitate different responses (i.e., in response alternation). In contrast to this, identical or similar stimuli that require the same response facilitate response selection, thus improving those measures (Colzato, Warrens, et al., 2006; Hommel, 2004). Similarly, differences between levels of feature overlap can also affect neurophysiological measures. For instance, the mean amplitude of the P3 event‐related potential and the small‐world network coefficient also show changes in opposite directions when associated with partial repetition cost or response facilitation, respectively (Takacs, Zink, et al., 2020). Thus, the outcome measure will not be confounded by the direction of binding‐associated changes in accuracy and RTs when only using the absolute values of the partial repetition cost and the response benefit. This quantification of binding effects corresponds to an earlier study (Petruo et al., 2016). However, please note that this nonstandard method does not differentiate between the levels of feature overlap based on the type of the overlapping dimension (i.e., colour, orientation, position) (Colzato, Warrens, et al., 2006). Finally, the obtained binding measure was correlated with GABA+/NAA levels.

For both the ANOVA and the Pearson correlations, the Bayes factor (*BF*_*01*_) is reported to quantify the evidence for the null hypothesis. For the ANOVAs, *BF*_01_ was calculated according to Masson (Masson, 2011), and for the correlations pairs, *BF*_01_ values were obtained from JASP. For descriptive statistics, the mean and standard error of the mean (SEM) are given.

## 3 RESULTS

### 3.1 Analyses of task effects

**FIGURE 5:**
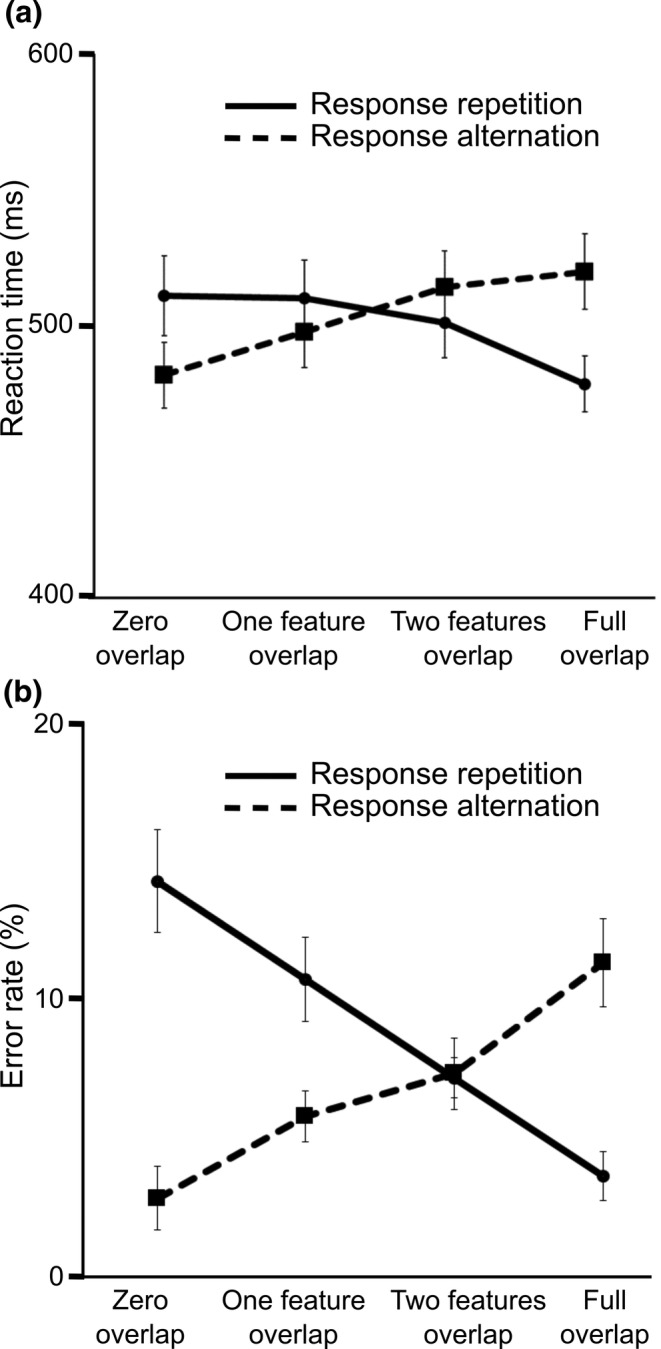
Interaction between stimulus and response features on the behavioural level. (a) Reaction times across feature overlap and response type conditions. The median RT is shown as a function of overlapping features for repeated and alternated responses. Repeated responses are indicated by solid lines, alternated responses are indicated by dotted lines. Error bars denote the standard error of mean. (b) Error rate results across feature overlap and response type conditions. The percentage of incorrect trials is shown as a function of overlapping features for repeated and alternated responses. Repeated responses are indicated by solid lines, alternated responses are indicated by dotted lines. Error bars denote standard error of mean

The feature overlap by response type ANOVA on the reaction time (RT) data showed that the main effects of feature overlap (*F*(3,102) = 1.86, *p* = .141, η_p_^2^ = .053, *BF*_01_ = 18.80) and response type (*F*(1,34) = 0.59, *p* = .447, η_p_^2^ = .018, *BF*_01_ = 4.33) were not significant. However, the interaction of feature overlap and response type was significant (*F*(3,102) = 22.71, *p* < .001, η_p_^2^ = .402, *BF*_01_ < 0.01), as shown in Figure 5a.

When responses had to be repeated, RTs were significantly shorter in the full overlap condition (480 ms ± 10) than in the zero overlap (514 ms ± 15, *p* = .005), the one feature overlap (513 ms ± 14, *p* = .002), and the two features overlap conditions (503 ms ± 13, *p* = .026). No other pairwise contrasts were significant in response repetition trials (all *p* > .05). When responses had to be alternated, RTs were faster in the zero overlap condition (484 ms ± 12) than in the two feature overlap (516 ms ± 14, *p* < .001) and the full overlap conditions (522 ms ± 14, *p* < .001). Additionally, participants responded faster in the one feature overlap condition (506 ms ± 14) than in the two features overlap condition (*p* = .002) when responses had to be alternated. No other pairwise contrasts were significant in response alternation trials (all *p* > .05).

The feature overlap by response type ANOVA on the error rate (see Figure 5b) showed that the main effect of feature overlap was not significant (*F*(3,102) = 1.17, *p* = .324, η_p_^2^ = .034, *BF*_01_ = 86.64). However, the main effect of response type (*F*(1,34) = 5.66, *p* = .023, η_p_^2^ = .146, *BF*_01_ = 0.40) was significant. Participants produced less errors for response alternation than for response repetition (6.8% ± 1.0 vs. 8.9% ± .9). The interaction of feature overlap and response type was also significant (*F*(3,102) = 27.01, *p* < .001, η_p_^2^ = .451, *BF*_01_ = 0.01). When responses had to be repeated, participants made less errors in the full feature overlap condition (3.8% ± 0.9) than in the zero (15.3% ± 1.8, *p* < .001), one feature (10.2% ± 1.4, *p* = .002), and two features overlap conditions (7.3% ± .7, *p* = .002). Additionally, error rate was lower in the one feature overlap condition than in the zero overlap condition (*p* = .009). When responses had to be alternated, participants made more errors in the full feature overlap (11.7% ± 1.5) than in the zero (3.2% ± 1.1, *p* < .001), one feature (5.5% ± 0.8, *p* < .001), and two features overlap conditions (7.8% ± 1.3, *p* = .029). Additionally, error rate was higher in the two features overlap than in the zero overlap condition (*p* = .029).

### 3.2 GABA+/NAA levels and overall binding effects

**FIGURE 6:**
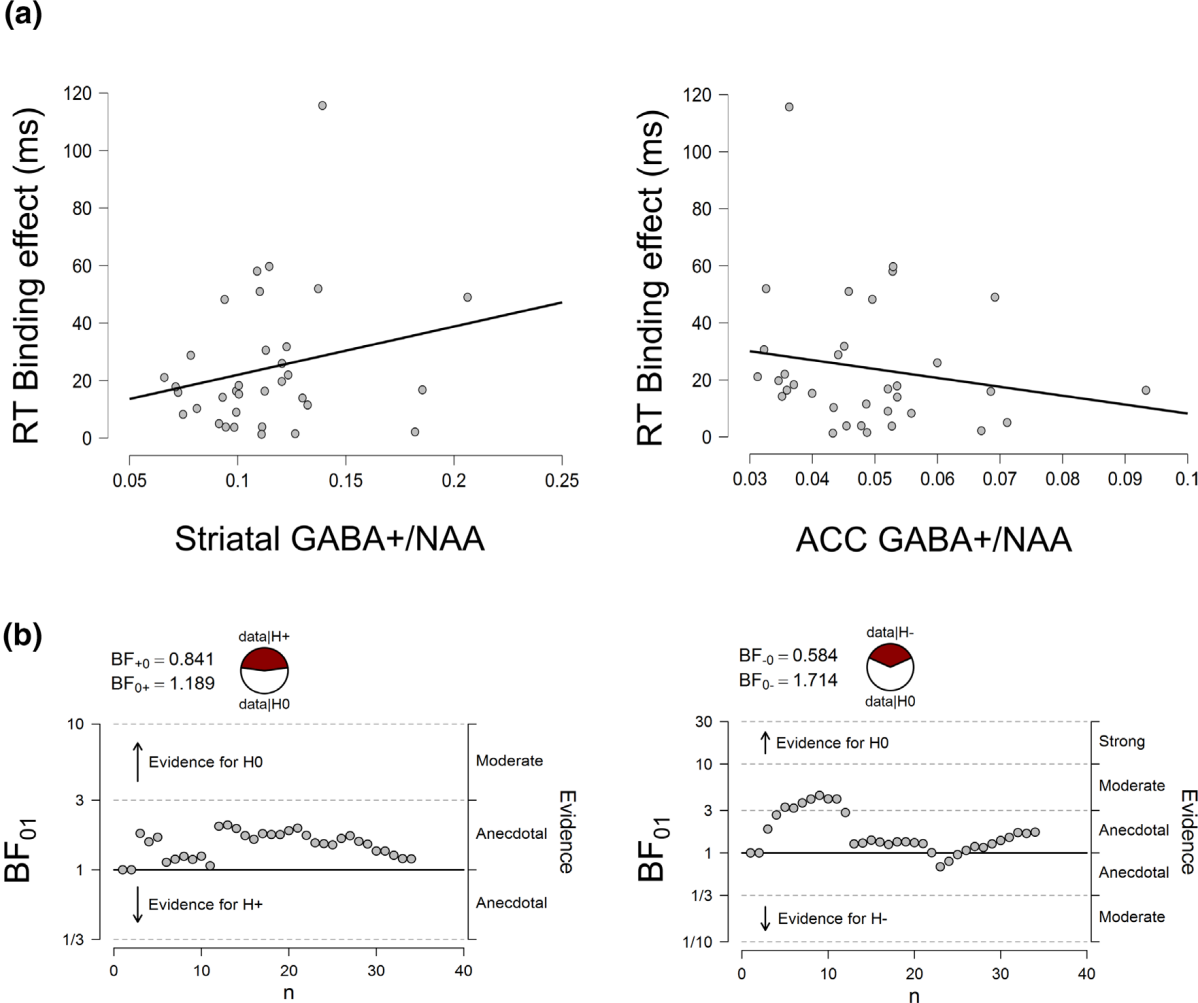
(a) Scatter plots denoting the correlation between the behavioural binding effect (absolute RT differences between full feature overlap and zero overlap) and the GABA+/NAA levels in the striatum (left graph) and in the ACC (right graph). (b) The sequential analyses display the development of the Bayes factor as the data accumulates over the sample. The display is based on directed hypothesis given the original direction of the correlation (i.e., positive or negative relationship). That is, evidence represented by individual subjects either contributes to the support for the null hypothesis (above the threshold line), or to the support of the alternative hypothesis (below the threshold line). The accumulated evidence is summarized as probabilities of the data given the null or the alternative hypothesis. Probability wheels are depicting the odds of the data under the null hypothesis (data|H0) vs. alternative hypothesis (data|H+/−)

The feature overlap by response type interaction was supported by both traditional statistical interference and Bayesian analysis both for the RT and error rate data. However, to reduce the number of correlations and type I errors, we decided to use the binding measure obtained from the RT data for correlation analyses with GABA+/NAA spectroscopy data. Plots illustrating the correlations between binding and ACC as well as striatal GABA+/NAA levels are provided in Figure 6.

The correlation between the GABA+/NAA concentration in the striatum and binding was not significant and the Bayesian analysis supported the null hypothesis (*r* = .226; *p* = .199; *BF*_01_ = 1.19). Thus, there was no substantial evidence for a functional relationship between striatal GABA+/NAA concentrations and event file binding. Similarly, ACC GABA+/NAA levels did not correlate significantly with binding and the Bayes factor also supported the null hypothesis (*r* = −.179; *p* = .312; *BF*_01_ = 1.71). Thus, there was no substantial evidence for a correlation between GABA+/NAA levels in the ACC and event file binding, either.

### 3.3 GABA+/NAA levels and binding effects in the early and late phases of the task

**FIGURE 7:**
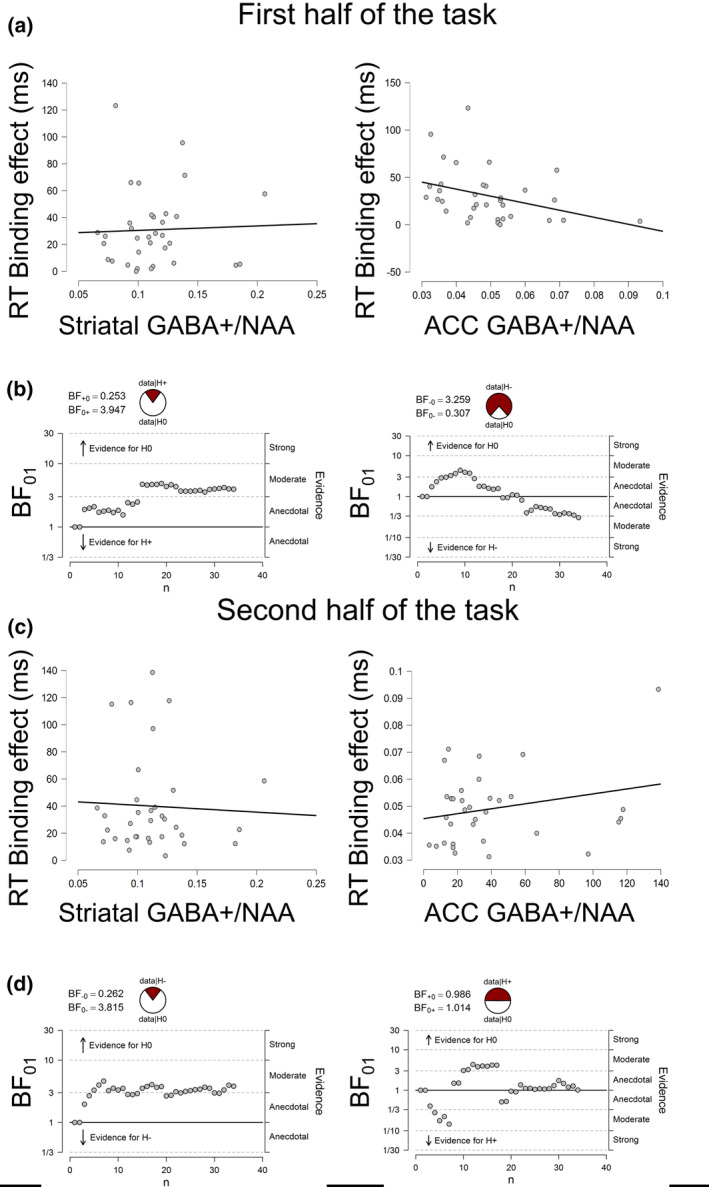
(a) Scatter plots denoting the correlation between the event file binding effect (absolute RT differences between full feature overlap and zero overlap) and the GABA+/NAA levels in the striatum (left graph) and in the ACC (right graph) in the first half of the task (trials 1 to 128). (b) The sequential analyses display the development of the Bayes factor as the data accumulates in the first half of the task. The display is based on directed hypothesis given the original direction of the correlation (i.e., positive or negative relationship). Evidence represented by individual subjects either contributes to the support for the null hypothesis (above the threshold line), or to the support of the alternative hypothesis (below the threshold line). The accumulated evidence is summarized as probabilities of the data given the null or the alternative hypothesis. Probability wheels are depicting the odds of the data under the null hypothesis (data|H0) vs. alternative hypothesis (data|H+/−). (c) Scatter plots denoting the correlation between the event file binding effect and the GABA+/NAA levels in the striatum (left graph) and the ACC (right graph) in the second half of the task (trials 129 to 256). (d) The sequential analyses display the development of the Bayes factor as the data accumulates in the second half of the task

To analyse the potentially different roles of striatal and ACC GABA+/NAA levels in different phases of the event file coding experiment, we split the experiment in a first and a second half, each of which contained 128 trials. We then performed separate correlation analyses for both halves of the task, which were otherwise analogous to the correlation analyses run over the entire task data. Correlations between these behavioural measures and ACC / striatal GABA+/NAA levels in the first and in the second half of the trials are provided in Figure 7.

In the first half of the task, the correlation between the striatal GABA+/NAA concentrations and binding was not significant and the Bayesian analysis provided moderate evidence for the null hypothesis (*r* = .038; *p* = .833; *BF*_01_ = 3.95). However, the correlation between ACC GABA+/NAA and binding was significant and the Bayesian analysis provided moderate evidence for the alternative hypothesis (*r* = −.357; *p* = .038; *BF*_01_ = 0.31). Specifically, higher ACC GABA+/NAA levels were associated with stronger binding effects, while the striatal GABA+/NAA system did not seem to be relevant in the first half of the task.

In the second half of the task, the correlation between striatal GABA+/NAA concentrations and binding was not significant and the Bayesian analysis provided moderate evidence for the null hypothesis (*r* = −.045; *p* = .802; *BF*_01_ = 3.82). Similarly, the correlation between ACC GABA+/NAA and binding was not significant and the Bayesian analysis provided evidence for the null hypothesis (*r* = .244; *p* = .164; *BF*_01_ = 1.01). In sum, the GABA+/NAA system does not seem to be related to event file binding in the second half of the task.

To confirm the changing role of ACC GABA+/NAA during the course of the task, we also investigated the correlation between learning/practice effect in binding, and ACC GABA+/ACC. That is, we calculated the difference in event file binding between the first and second half of the task. This learning effect in event file binding correlated significantly with ACC GABA+/NAA and the Bayesian analysis supported the alternative hypothesis (*r* = −.384; *p* = .025; *BF*_01_ = 0.21). Thus, task experience is an important factor for the relevance of the ACC GABA+/NAA in event file binding. Striatal GABA+/NAA, which had previously not been shown to correlate with binding in either parts of the task, also did not significantly correlate with the learning effect and the Bayesian analysis provided moderate evidence for the null hypothesis (*r* = .054; *p* = .761; *BF*_01_ = 3.64). In sum, ACC (but not striatal) GABA+/NAA modulates task experience effects in event file binding.

## 4 DISCUSSION

In the current study, we examined the neurobiological underpinnings of response selection and event file binding mechanisms with a focus on the functional role of the striatal and the medial frontal (ACC) GABAergic system. To the best of our knowledge, this is the first study examining structure‐specific effects of the GABAergic system on stimulus–response binding in event file coding in human subjects. We employed a standard event file task (Colzato, van Wouwe, Lavender, & Hommel, 2006), in which the stimulus feature overlap of two successively presented stimuli and the required responses are systematically varied. At the behavioural level, participants were the fastest and most accurate when they needed to repeat their responses in the context of repeating stimulus features. However, participants became slower and less accurate in the same context of stimulus feature repetition when their response needed to be altered (partial repetition cost). This evidences that the participants' behaviour was modulated by the formation of event files as suggested by the TEC: pre‐established bindings facilitated responding, while unbinding caused the opposite effect (Colzato, Warrens, et al., 2006; Hommel, 2004).

Based on previous studies of response selection and maintaining task representations, we envisaged two possible scenarios of how inter‐individual differences in the MRS data could reflect in these behavioural effects. The first possibility, which was directly measured and tested in our study, postulated that higher GABA levels facilitate event file coding based on the winner‐take‐all processing of predominant response selection (Haag et al., 2015; Kühn et al., 2011; Quetscher et al., 2015; Yildiz et al., 2014). As a second possibility, an alternative account links the dopaminergic, and not the GABAergic, system to event file coding (Colzato et al., 2012, 2013, 2016). While this alternative explanation was not directly tested in the current study, the obtained evidence against the correlation between binding effects and GABA levels in the striatum / ACC could still lend some support to such potential dopaminergic explanations.

Importantly, behavioural binding effects (as assessed via the average absolute difference between no feature overlap and full feature overlap conditions) were not correlated with the GABA+/NAA concentration either in the striatum, or in the ACC. Bayesian analyses further confirmed the independence of binding and GABA+/NAA levels. In line with these findings, another study from our lab recently found that event file coding seems to be unaltered during alcohol high‐dose intoxication (Stock et al., 2019), which is known to strongly modulate GABAergic signalling in fronto‐striatal circuits (Iversen, Iversen, Bloom, & Roth, 2009). Based on this, the authors proposed that event file coding is likely not modulated by fronto‐striatal GABA (Stock et al., 2019). Yet, this argument remained tentative, as alcohol also affects various other neurobiochemical systems, including glutamatergic, dopaminergic, and serotonergic signalling (Chastain, 2006). Against this background, the current study provides further evidence that event file processes are not generally dependent on GABA levels in brain regions that are commonly considered to be functionally relevant (i.e., the striatum and/or the ACC).

Still, this lack of effects may be seen as being at odds with previous models suggesting that striatal GABA plays a key role in integrating and selecting different downstream action requests (Bahuguna, Weidel, & Morrison, 2019; Buxton, Bracci, Overton, & Gurney, 2017; Tomkins et al., 2014). Similarly, previous studies proposed that event file coding depends on the striatum (Colzato et al., 2016; Persson et al., 2015). When it comes to maintaining task representations, however, the dopaminergic system seems to be of greater functional importance and relevance than the GABAergic system (Durstewitz & Seamans, 2008; Seamans & Yang, 2004). Indeed, genotypes that significantly influence the amount of striatal dopamine (Colzato et al., 2016) or striatal dopamine receptor density (Persson et al., 2015) are related to the updating of mental sets. Moreover, striatal dopamine levels modulate the control of stimulus–response associations (Colzato et al., 2013) and increased striatal dopamine concentrations in L‐dopa‐medicated Parkinson's patients have been shown to lead to enhanced event file binding (Colzato et al., 2012). Based on this, a dissociated pattern may be envisaged, where striatal GABA can determine response selection processes, while striatal dopamine plays a role in response and stimulus integration as well as the configuration of event files. Another potential explanation could lie in the nature of the task. Striatal MSNs actively inhibit action requests that would cause disordered motor activations (Buxton et al., 2017; Tomkins et al., 2014). This selection process can best be observed in action sequences, where the orchestration of subsequent actions is pivotal. There is evidence for the causal role of GABA in sequence learning (Jongkees, Immink, & Colzato, 2017) and the chunking of action sequences seems to be a central computational function modulated by striatal GABA (Buxton et al., 2017). However, event file coding does not require extensive chunking of action sequences, as responses are dependent on bindings that already exist. However, these associations change dynamically through unbinding and they do not form longer sequences. It is hence possible that a more complex sequential structure is needed for the involvement of striatal GABA signalling.

While ACC GABA+/NAA did not correlate with event file binding in the entire task, it was related to task performance in the first half of the event file coding paradigm. That is, higher GABA+/NAA concentrations in the ACC were correlated with stronger event file binding (i.e., partial repetition cost) in the beginning of the task. As participants gathered more experience with the task, this relationship waned so that ACC GABA+/NAA did no longer correlate with stronger event file bindings in the second half of the task. This dissociation was further confirmed by the correlation between the task's practice/learning effect (reflected by the change in binding between the first and second halves of the task) and ACC GABA+/NAA levels. Crucially, time on task and the associated learning of feature properties affects how well they can be bound, which can either increase, or completely eliminate the binding effect (Colzato, Raffone, & Hommel, 2006; Colzato, Warrens, et al., 2006; Hommel, 2009; Hommel & Colzato, 2009). Especially in case of artificial stimuli, increasing practice might actually decrease event file binding (Colzato, Raffone, et al., 2006), but this decrease was only evident for irrelevant bindings. That is, binding effects of response‐relevant stimulus features seemed to be preserved (e.g., between shape and location) while binding effects decrease for response‐irrelevant stimulus features (e.g., between colour and location) (Colzato, Raffone, et al., 2006). Other studies also reported decreasing bindings for irrelevant associations (Keizer, Verment, et al., 2010; Keizer, Verschoor, et al., 2010). It was suggested that the attenuation of response‐irrelevant bindings occurs as participants learn not to retrieve the task‐irrelevant bindings. Thus, this effect is more of a control or attention‐related effect rather than learning per se. This argument is in line with the notion that higher intelligence is correlated with a reduction in retrieving irrelevant bindings (Colzato, van Wouwe, et al., 2006). Hence, it seems plausible that ACC GABA+/NAA might only play a relevant role for S‐R‐binding tendencies before we learn to effectively distinguish between relevant and irrelevant stimuli / stimulus features. However, it is worth to note that similar task dynamics have been reported in procedural sequence learning. Namely, participants learn to implicitly allocate less attention to stimuli which are not relevant in sequence learning contexts (Janacsek & Nemeth, 2012; Song, Howard, & Howard, 2007; Takács et al., 2018). Thus, changes in attention allocation to certain stimulus categories can be a result of learning or practice effects. However, the potential link between inter‐individual differences in GABA levels and task‐relevant vs. task‐irrelevant bindings could not be investigated in the current study, as event file coding had been quantified as the absolute difference between feature overlap levels, irrespective of the type of overlap. Moreover, limiting the analyses to task‐relevant bindings would have resulted in insufficient trial numbers per participant. Therefore, future studies will be required to separately investigate the role of GABA levels in task‐relevant and task‐irrelevant bindings.

Although GABAergic interneuron density is particularly high in the striatum (Bernácer, Prensa, & Giménez‐Amaya, 2012), GABAergic neurons are also abundant in cingulate areas (Whissell, Cajanding, Fogel, & Kim, 2015), where they are central for response selection and control mechanisms (Silveri et al., 2013). Interestingly, electrophysiological and computational evidence (Adams, Sherfey, Kopell, Whittington, & LeBeau, 2017) suggests that the ACC GABA system is important to select (route) or combine information to efficiently respond to specific inputs (Adams et al., 2017). Such a dual role of the GABA system has not been put forward for the striatum, which has predominantly been associated with response selection. Yet, it may explain why ACC GABA+/NAA concentrations were correlated with partial repetition costs in the first half of the task. This result strengthens the notion that the ACC is implicated in response conflict situations, that is, when there is an interference or interaction between information processing pathways (Becker, Prat, & Stocco, 2016; Braver, Barch, Gray, Molfese, & Snyder, 2001). At the same time, it also strengthens the notion that the ACC is specifically related to response selection (Dalley et al., 2004; Rushworth et al., 2007; Shenhav et al., 2018). Furthermore, the current result is in line with previous research showing a link between the ACC GABA system and response modulation processes (Silveri et al., 2013). It has been shown that GABA levels in the ACC were positively associated with better response inhibition and lower impulsivity. Thus, higher GABA+ in the ACC is related to better behavioural control, possibly due to more adaptive response selection. Similarly, in the current study, higher ACC GABA+/NAA was positively correlated with better event file binding, that is, with a stronger stimulus–response association. Please note that in the S‐R task, event file binding is considered as an adaptive mechanism, contrary to other forms, such as distractor‐response binding (Opitz et al., 2020). In the current study, we dovetailed this concept by providing evidence that ACC is involved in event file binding, which is however modulated by practice.

Lastly, it should not go unmentioned that even though we did not find any literature suggesting sex differences in event file coding and decided exclude females because changes in steroid hormones across the menstrual cycle have been suggested to modulate GABA levels (Epperson et al., 2006; Harada et al., 2011) and would thus increase between‐subject variance, it would have been more representative to assess our research question in a mixed‐sex sample. It might also have allowed for conclusions on whether and how sex differentially modulates the effects of amino acid transmitters in the ACC and striatum onto event file coding. Some studies report that females have reduced GABA+ levels in the dorsolateral prefrontal cortex as compared to males (O'Gorman et al., 2011), which might potentially be linked to a lower GABA_A_ receptor α3 subunit expression in females than in males (Pandya et al., 2019). Epperson et al. (2002) suggested that healthy women with a natural menstrual cycle experience a decline in cortical GABA across the menstrual cycle, whereas De Bondt et al. (2015) reported a significant increase in prefrontal GABA during ovulation. Additionally, it has furthermore been suggested that this pattern might differ in women with premenstrual dysphoric disorder (Bäckström et al., 2011; De Bondt et al., 2015). To further complicate the matter, De Bondt et al. (2015) reported finding no prefrontal GABA differences between the pill phase and the non‐pill phase (as opposed to the changes seen in naturally cycling women), but Müller et al. (2020) reported that they did not find any effects of hormonal contraception on prefrontal GABA+ concentrations. Given that these findings are typically based very small samples (often less than 10 women per group), there is not yet a conclusive answer to how women might differ from men in terms of GABA+ levels and which factors (menstrual cycle, hormonal contraception, age, comorbid disorders etc.) could further modulate or mask those effects. Given that our sample was not representative of both sexes and that there is not yet enough data available to conclusively determine whether or not GABA levels would be functionally different between the sexes, the generalizability of our findings to the entire population remains to be investigated in future studies.

### 4.1 Conclusion

We examined structure‐specific effects of the GABAergic system on event‐file‐associated stimulus–response binding with a focus on the functional role of the striatal and the medial frontal (ACC) systems. These two systems showed distinctive patterns of behavioural associations: While striatal GABA+/NAA was independent from event file binding, higher GABA+/NAA levels in the ACC were related to stronger event file binding. However, the link between ACC GABA+/NAA and stimulus–response binding waned as the task progressed, which suggests that practice and learning effects can modulate the role of medial frontal GABA in binding processes.

## CONFLICT OF INTEREST

The authors report no conflicts of interest.

## Supporting information


**Data S1.** Supplementary Information.
**Figure S1.** Striatal GABA+/Glx and binding in the first half of the task Scatter plot illustrating the correlation between the behavioural binding effect (absolute RT differences between full feature overlap and zero overlap) in the first half of the task and the GABA+/Glx levels in the striatum.
**Table S1.** Comprehensive list and descriptive data of all measured transmitters and metabolites reported in the results section of the main text. Correlations with event file binding in the whole task are provided

**Video S1.** Supplementary Information.

## Data Availability

The anonymized data reported in the study and the associated data analysis codes can be made available for other researchers upon request sent to the corresponding authors. This procedure complies with the requirements of our funding agents and the institutional ethics approval.
