# Supplementary material for: On the functional role of striatal and anterior cingulate GABA+ in stimulus‐response binding
Source: Hum Brain Mapp. 2021 Jan 9;42(6):1863–78. doi: 10.1002/hbm.25335 (PMC7978129; doi:10.1002/hbm.25335)
Supplement: Supplementary file 1 — Data S1. Supplementary Information. Figure S1. Striatal GABA+/Glx and binding in the first half of the task Scatter plot illustrating the correlation between the behavioural binding effect (absolute RT differences between full feature overlap and zero overlap) in the first half of the task and the GABA+/Glx levels in the striatum. Table S1. Comprehensive list and descriptive data of all measured transmitters and metabolites reported in the results section of the main text. Correlations with event file binding in the whole task are provided [file HBM-42-1863-s001.pdf]

Supplementary Information for

On the functional role of striatal and anterior cingulate GABA+ in stimulus-response binding

Adam Takacs, Ann-Kathrin Stock, Paul Kuntke, Annett Werner, Christian Beste

Supplementary Table 1. Comprehensive list and descriptive data of all measured transmitters and metabolites reported in the results section of the main text. Correlations with event file binding in the whole task are provided.

	Striatal GABA+	Striatal tCr	Striatal NAA	ACC GABA+	ACC tCr	ACC NAA	Striatal Glu*	Striatal tCr*	ACC Glu*	ACC tCr*
Mean	430.12	3036.47	4035.29	286.22	3770.88	5859.41	1.70	1.57	2.02	1.92
SD	80.16	374.21	613.45	92.54	658.39	1034.07	0.24	0.16	0.34	1.46
r	.111	-.185	-.220	-.298	-*.363*	-.286	.052	-.159	.021	-.112
p	.532	.414	.212	.086	*.034*	.100	.772	.368	.911	.541
BF_{01}	3.86	2.77	2.22	1.14	*0.55*	1.29	4.50	3.17	4.52	3.80

*Edit off acquisition.

Supplementary Results

The LC model software package [Provencher, 1993], which was used for quantifying the transmitter and metabolite concentrations reported in the main text, did not allow to control for the fractions of grey matter (GM) and white matter (WM) in the individual voxels. For this reason, we additionally quantified transmitters and metabolites using the toolbox provided by Gannett et al. [Edden et al., 2014], which provides a reliable and relatively easy-to-obtain quantification of GM, WM, and cerebrospinal fluid (CSF) in the measured VOIs. Based on the obtained data, we ran additional analyses that also accounted for this factor (see below).

Glx/NAA levels and binding effects

The fractions of GM, WM and CSF in the ACC did not correlate with the levels of Glx and NAA measured for this VOI ($p > .092$). Similarly, the fractions of GM, WM and CSF in the striatum did not correlate with the levels of Glx and NAA in these VOIs ($p > .112$).

The correlation between Glx/NAA concentrations in the striatum and binding effects over the whole task was not significant ($r = .143$; $p = .214$; $BF_{01} = 2.19$). However, the correlation between Glx/NAA concentrations in the ACC and overall binding effects was significant ($r = .325$; $p = .033$; $BF_{01} = 0.47$), suggesting greater binding effects in case of higher Glx/NAA concentrations in the ACC.

In the first half of the task, the correlation between Glx/NAA concentrations and binding was not significant for either the striatum ($r = -.074$; $p = .682$; $BF_{01} = 4.26$) or the ACC ($r = .271$; $p = .127$; $BF_{01} = 1.51$). In the second half of the task, the correlation between Glx/NAA concentrations and binding effects was also not significant for the striatum ($r = .149$; $p = .408$; $BF_{01} = 3.33$) or the ACC ($r = -.161$; $p = .371$; $BF_{01} = 3.15$).

To investigate the changing role of Glx/NAA during the course of the task, we also analysed the correlation between learning/practice effect in binding, and striatal Glx/NAA. That is, we calculated the difference in event file binding between the first and second half of the task. This learning effect in event file binding did not significantly correlate with Glx/NAA in the striatum ($r = -.150$; $p = .406$; $BF_{01} = 3.31$) or in the ACC ($r = .270$; $p = .129$; $BF_{01} = 1.53$).

GABA+/Glx levels and binding effects

Given that GABA and glutamate have opposing inhibitory vs. excitatory effects on neurons, we further ran exploratory add-on analyses with the GABA+/Glx ratio. For the correlations with fractions of GM, WM and CSF, please see above and in the main text (2.3 MRS data acquisition and processing). The correlation between GABA+/Glx concentration and binding in the whole task was not significant for the striatum (r = .273; p = .131; BF_{01} = 1.53) or for the ACC (r = -.024; p = .897; BF_{01} = 4.51).

In the first half of the task, the correlation between striatal GABA+/Glx concentrations and binding effects was significant (r = .383; p = .031). Bayesian analysis provided moderate evidence for the positive correlation (BF_{01} = 0.25). This means that higher GABA+ relative to Glx concentration was related to stronger event file binding in the beginning of the task (see Supplementary Figure 1).

However, the correlation between ACC GABA+/Glx and binding in the first half of the task was not significant (r = .034; p = .854; BF_{01} = 4.48). In the second half of the task, the correlation between GABA+/Glx concentrations and binding was not significant for the striatum (r = -.118; p = .519; BF_{01} = 3.73) or for the ACC (r = .131; p = .473; BF_{01} = 3.56).

To investigate the changing role of GABA+/Glx during the course of the task, we also analysed the correlation between learning/practice effects in binding and the GABA+/Glx ratio. This did not yield significant correlations for the striatum (r = .301; p = .094; BF_{01} = 1.19) or for the ACC (r = -.079; p = .668; BF_{01} = 4.17).

Supplementary Figure 1. Striatal GABA+/Glx and binding in the first half of the task

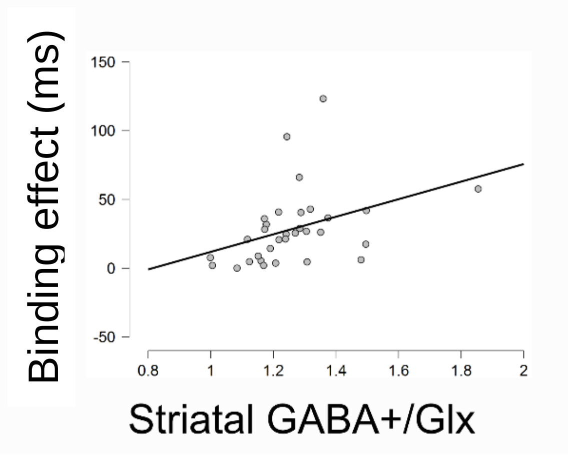

Scatter plot illustrating the correlation between the behavioural binding effect (absolute RT differences between full feature overlap and zero overlap) in the first half of the task and the GABA+/Glx levels in the striatum.

References

Edden RAE, Puts NAJ, Harris AD, Barker PB, Evans CJ (2014): Gannet: A batch-processing tool for the quantitative analysis of gamma-aminobutyric acid–edited MR spectroscopy spectra. Journal of Magnetic Resonance Imaging 40:1445–1452.

Provencher SW (1993): Estimation of metabolite concentrations from localized in vivo proton NMR spectra. Magn Reson Med 30:672–679.
